# Concentrations of Urinary Phthalate Metabolites Are Associated with Increased Waist Circumference and Insulin Resistance in Adult U.S. Males

**DOI:** 10.1289/ehp.9882

**Published:** 2007-03-14

**Authors:** Richard W. Stahlhut, Edwin van Wijngaarden, Timothy D. Dye, Stephen Cook, Shanna H. Swan

**Affiliations:** 1 Department of Community and Preventive Medicine, University of Rochester School of Medicine and Dentistry, Rochester, New York, USA; 2 Department of Research and Evaluation, Axios International, Paris, France; 3 Department of Pediatrics and; 4 Department of Obstetrics and Gynecology, University of Rochester School of Medicine and Dentistry, Rochester, New York, USA

**Keywords:** androgens, homeostatic model assessment, insulin resistance, obesity, phthalates

## Abstract

**Background:**

Phthalates impair rodent testicular function and have been associated with anti-androgenic effects in humans, including decreased testosterone levels. Low testosterone in adult human males has been associated with increased prevalence of obesity, insulin resistance, and diabetes.

**Objectives:**

Our objective in this study was to investigate phthalate exposure and its associations with abdominal obesity and insulin resistance.

**Methods:**

Subjects were adult U.S. male participants in the National Health and Nutrition Examination Survey (NHANES) 1999–2002. We modeled six phthalate metabolites with prevalent exposure and known or suspected antiandrogenic activity as predictors of waist circumference and log-transformed homeostatic model assessment (HOMA; a measure of insulin resistance) using multiple linear regression, adjusted for age, race/ethnicity, fat and total calorie consumption, physical activity level, serum cotinine, and urine creatinine (model 1); and adjusted for model 1 covariates plus measures of renal and hepatic function (model 2). Metabolites were mono-butyl phthalates (MBP), mono-ethyl phthalate (MEP), mono-(2-ethyl)-hexyl phthalate (MEHP), mono-benzyl phthalate (MBzP), mono-(2-ethyl-5-hydroxyhexyl) phthalate (MEHHP), and mono-(2-ethyl-5-oxohexyl) phthalate (MEOHP).

**Results:**

In model 1, four metabolites were associated with increased waist circumference (MBzP, MEHHP, MEOHP, and MEP; *p*-values ≤ 0.013) and three with increased HOMA (MBP, MBzP, and MEP; *p*-values ≤ 0.011). When we also adjusted for renal and hepatic function, parameter estimates declined but all significant results remained so except HOMA-MBP.

**Conclusions:**

In this national cross-section of U.S. men, concentrations of several prevalent phthalate metabolites showed statistically significant correlations with abdominal obesity and insulin resistance. If confirmed by longitudinal studies, our findings would suggest that exposure to these phthalates may contribute to the population burden of obesity, insulin resistance, and related clinical disorders.

Obesity, insulin resistance, and type 2 diabetes are interrelated metabolic disorders whose prevalence has increased substantially in the past two decades. Corresponding increases in premature morbidity and mortality are expected (Adams et al. 2006; Fujimoto 2000; Haffner et al. 1998; Poirier et al. 2006; Zimmet et al. 2001). Insulin resistance occurs when increasing amounts of insulin are required to correctly regulate transport of plasma glucose into peripheral tissues. Although the precise mechanism is unclear, insulin resistance is commonly accompanied by central (visceral) obesity, which, by elevating levels of free fatty acids in serum, may provoke insulin resistance and disrupt lipid metabolism. Initially, the beta cells of the pancreas can fully compensate for mild insulin resistance by increasing insulin production. As the disease progresses, beta cells decompensate, resulting in elevated serum glucose levels and the subsequent development of type 2 diabetes.

Testosterone affects body fat distribution and insulin sensitivity in men. Experimental studies in males have shown that testosterone administration reduces lipid uptake by intraabdominal fat (Mårin et al. 1996) and also reduces visceral fat and improves insulin sensitivity (Mårin 1995; Mårin et al. 1992, 1993). A 2005 meta-analysis found that testosterone administration reduces total fat mass (Isidori et al. 2005). Men undergoing androgen deprivation therapy for prostate cancer have increased serum glucose, total fat, and prevalence of metabolic syndrome (Braga-Basaria et al. 2006; Sharifi et al. 2005). Epidemiologic studies often support these findings (Ding et al. 2006; Selvin et al. 2007), but sometimes they do not (Oh et al. 2002).

Humans are commonly exposed to man-made chemicals that have the potential to reduce androgen (e.g., testosterone) production or function. One such class of chemicals is phthalates, which are used in a variety of products, including cosmetics, shampoos, soaps, lubricants, pesticides, and paints; it is also used as a softener of polyvinyl chloride. More than 75% of the U.S. population has measurable levels of several phthalate metabolites in the urine (Silva et al. 2004). Unlike polychlorinated biphenyls (PCBs) and dioxins, phthalates are quickly metabolized and excreted (Hauser and Calafat 2005). The half-life of di(2-ethylhexyl)phthalate (DEHP), one of the most widely used and studied phthalates, is < 24 hr (Koch et al. 2004).

Phthalates are known antiandrogens in experimental animal models, with consistent results dating back several decades. Testicular steroid hormone synthesis and reproductive system development in males have been adversely affected by exposure, especially neonatal exposure, to certain phthalates, including DEHP, di-butyl phthalate (DBP), benzyl-butyl phthalate, and di-isononyl phthalate (Bell 1982; Fisher 2004; Parks et al. 2000).

Associations between certain phthalate metabolites and antiandrogenic effects have also been found in humans at much lower exposure levels than those used in rodent experiments. Suspected metabolites include mono-benzyl phthalate (MBzP), mono-ethyl phthalate (MEP), mono-isononyl phthalate (MiNP), mono-methyl phthalate, and mono-butyl phthalate (MBP). Urinary phthalate metabolites in pregnant women have been found to correlate with subtle genital changes in their infant males (Swan et al. 2005), and breast-milk phthalate metabolites have been correlated with shifts in reproductive hormones in infant males (Main et al. 2006).

Although fetuses and infants are thought to be more susceptible to environmental insult than adults, Duty et al. (2003) and Hauser et al. (2006) found diminished sperm quality associated with urinary phthalate metabolites in adult males as well. If their findings reflect true antiandrogenic effects of phthalates or their metabolites at current exposure levels, then one may reasonably predict that these exposures could increase the prevalence of metabolic disorders that are worsened by diminished androgen production or function.

In this study we examined the association between phthalate exposure and two key metabolic abnormalities associated with hypoandrogenism: abdominal obesity and insulin resistance. Although these conditions are closely related, they represent key precursors—alone or in combination—to the development of type 2 diabetes and cardiovascular disease (Janssen et al. 2004; Reaven 1988). Our hypothesis was that increased phthalate exposure would be associated with increased abdominal obesity and insulin resistance.

## Methods

### Study population

We used data from the 1999–2002 National Health and Nutrition Examination Survey (NHANES) for this analysis. NHANES, conducted by the National Center for Health Statistics (NCHS), Centers for Disease Control and Prevention (CDC), is a multistage, stratified, clustered design that selects a representative sample of the civilian, noninstitutionalized U.S. population. Certain subgroups, such as older adults, Mexican Americans, non-Hispanic blacks, and low-income persons, were sampled at a higher rate than other demographic groups, thus necessitating the use of sample weights in analysis. Data from NHANES subjects are acquired through household interviews and standardized examinations at mobile examination centers throughout the United States. Detailed methods have been published elsewhere (NCHS 2006b).

We limited our analysis to men > 18 years of age with complete data for the measures described below. Men on insulin, oral hypoglycemic agents, or sex hormone agonists/antagonists were excluded because these medications may affect the biological mechanisms of interest. Men were also excluded from the insulin resistance analyses if they reported to NCHS that they failed to fast for 8–24 hr before collection of fasting blood samples.

In NHANES 1999–2002, a random one-third subsample was selected for urinary phthalate metabolite measurements, and a separate random, but overlapping, one-third subsample was selected for fasting glucose and insulin (used to compute insulin resistance) measurements. Of the 5,094 adult men available in NHANES, 1,451 men had both phthalate and obesity measurements for crude analyses after exclusions. Of these men, 45% (*n* = 651) also had fasting measurements. Two of the phthalate metabolites, mono-(2-ethyl-5-hydroxyhexyl) phthalate (MEHHP) and mono-(2-ethyl-5-oxohexyl) phthalate (MEOHP), were only available for the years 2001–2002, reducing sample size for these metabolites by about half (waist circumference, *n* = 781; insulin resistance, *n* = 344). Missing data reduced sample sizes for fully adjusted models (vs. crude) by 11% for waist circumference and 4.5% for insulin resistance.

### Abdominal obesity

Waist circumference was chosen as the best available measure of abdominal obesity. As a predictor of insulin resistance, waist circumference has been found to be an equivalent and, in some cases, a better measure than body mass index (Farin et al. 2006; Janssen et al. 2004). Waist circumference was measured at the high point of the iliac crest at minimal respiration to the nearest 1 mm.

### Insulin resistance

We estimated insulin resistance using HOMA (homeostatic model assessment). HOMA is epidemiologically practical, widely used, and correlates acceptably (*R* = 0.73–0.88) with the hyperinsulinemic-euglycemic clamp test, which is generally considered to be the gold standard (Matthews et al. 1985; Wallace et al. 2004). HOMA was calculated from fasting plasma glucose and insulin measures following the method of Matthews et al. (1985):


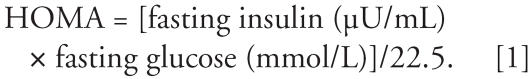


Plasma glucose was determined by an enzymatic reaction (Cobas Miras assay); plasma insulin was determined using a radio-immunoassay with the double-antibody batch method.

### Phthalate exposure

Phthalate data were collected in NHANES as urinary metabolites—rather than unmetabolized phthalates in serum—to eliminate contamination during collection and analysis (Latini 2005). Laboratory methods have been previously described (Silva et al. 2004). Seven metabolites were measured throughout the 4-year period, with five additional metabolites measured in 2001–2002 subjects only. For measurements below the limit of detection (LOD), the NCHS assigned a default value of LOD divided by the square root of 2, a method of handling nondetectable values that produces reasonably nonbiased means and SDs (Hornung and Reed 1990).

Of the seven phthalates with data for all 4 years, four (MBP, MEP, MiNP, MBP) are suspected human antiandrogens based on existing human studies (Duty et al. 2003; Hauser et al. 2006; Main et al. 2006; Swan et al. 2005). Metabolites of DEHP, the most widely studied antiandrogenic phthalate, were also of interest: mono(2-ethylhexyl) phthalate (MEHP) because it was available for all 4 years, and MEHHP and MEOHP (2001–2002 only) because secondary DEHP metabolites are suspected to be more biologically active (Koch et al. 2005; Stroheker et al. 2005). MiNP was eliminated from further consideration because its concentration was < LOD in > 75% of subjects. In NHANES 1999–2000, MBP represented both mono-*n*-butyl phthalate and mono-isobutyl phthalate, whereas in NHANES 2001–2002, these metabolites were measured separately (CDC 2005). In the present study, we summed the two mono-butyl phthalate metabolites in 2001–2002 data to permit analyses of mono-butyl phthalates over the 4-year period.

### Potential confounders

Covariates included in our analyses were age, race/ethnicity, family history of diabetes, dietary fat and caloric intake, physical activity, income, renal function, hepatic function, and exposure to tobacco smoke. Race/ethnicity was self-identified (white, black, Mexican American, other Hispanic, and other/multiethnic). Family history of diabetes was dichotomous (yes, no). Total dietary fat (continuous) and caloric intake (continuous) were computed from a 24-hr recall dietary questionnaire. Two physical activity measures were included: moderate to vigorous leisure activity (continuous; metabolic equivalents/month) and video-based (computer, video, TV) inactivity (categorical, hours/day: 0, < 1, 1, 2, 3, 4, ≥ 5). Socioeconomic status was represented as a “poverty income ratio,” a measure of income relative to family size and compared to the federal poverty threshold (categorical, percentage of poverty threshold: < 100, 100–199, 200–299, 300–399, 400–499, > 500). The renal function measure was glomerular filtration rate (GFR; continuous), estimated using the four-variable equation from the Modification of Diet in Renal Disease study (Levey et al. 1999), incorporating serum creatinine adjustments per NCHS instructions (NCHS 2006c). Liver function was represented by alanine aminotransferase (ALT; continuous) and gamma glutamyl transferase (GGT; continuous). Exposure to tobacco smoke was determined using serum cotinine (continuous).

### Statistical analysis

For descriptive analyses, we computed median and mean phthalate metabolite levels, adjusting for urine concentration by dividing metabolite measurements by urine creatinine. These analyses were conducted for the entire sample, and also stratified by race/ethnicity and age.

Linear regression analyses were performed with HOMA (log-transformed) and waist circumference as outcome variables. Because several phthalate metabolites are strongly correlated, each urinary phthalate metabolite was examined separately. Phthalate metabolite concentrations were log-transformed to normalize the data. Categorical analyses by exposure quintiles were also performed.

We conducted crude analyses and two adjusted analyses for each phthalate. Adjusted model 1 includes covariates discussed below. Adjusted model 2 includes these covariates, plus GFR, ALT, and GGT. Model 1 was our primary model because phthalates are known to affect the liver (Bhattacharya et al. 2005; Lapinskas et al. 2005; Rusyn et al. 2006); therefore, adjusting for liver function (model 2) could remove true effects. Model 2 is also needed, however, because obesity can affect liver function (Lawlor et al. 2005) and thus alter phthalate metabolism. GFR had minimal effect on results but was left in model 2 for completeness.

Age and race/ethnicity are known confounders and were included in fully adjusted models. Urine creatinine was included to correct for urine concentration, as recommended by Barr et al. (2005). Because the relationship between age and prevalence of metabolic syndrome (an outcome related to our outcomes) appears curvilinear (Park et al. 2003), age^2^ was also included. Age, age^2^, urine creatinine, and race/ethnicity were forced into all models regardless of their influence on metabolite regression coefficients. Other covariates were evaluated as possible confounders.

Covariates whose removal caused metabolite parameter estimates to change by ≥ 10% were considered confounders and left in the adjusted models (Greenland 1989). Food intake (total fat and calories), serum cotinine, and activity measures were confounders for two or more metabolites, and were therefore included as covariates. Family history of diabetes and poverty income ratio were not important confounders and had many missing values; thus they were omitted. The sample size for the full regression model was reduced by 11% for waist circumference and 4.5% for insulin resistance due to missing data for one or more covariates.

To assess the contribution of phthalate metabolites to the model fit, the percent variation in the outcome measures explained by each metabolite was calculated by computing the difference in adjusted *R*^2^ between the full model with and without that metabolite.

To convert regression coefficients to clinically interpretable measures, we first computed the absolute change in waist circumference and log HOMA represented by an increase in the significant (*p* ≤ 0.05) log phthalate metabolites from the 10th to 90th percentiles, then calculated this increase in waist circumference and HOMA as a percent of their medians:


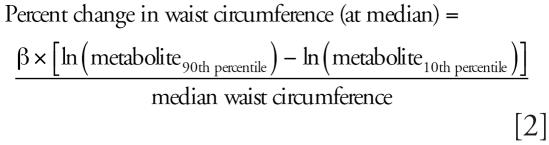



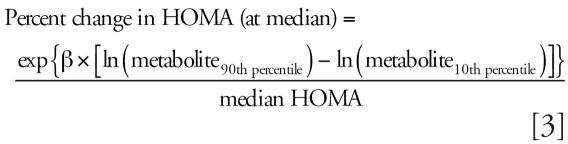


where β is the regression coefficient for each individual metabolite.

We used SAS, version 9.1 (SAS Institute, Cary, NC) for all statistical analyses. Appropriate weight variables were used to account for oversampling of special demographic groups in NHANES, and sampling cluster variables were used for its complex sampling design. Phthalate subsample weight variables were used for both waist circumference and HOMA analyses. Because we examined only the adult male subpopulation in the NHANES data, we used subpopulation methods as described by Graubard and Korn (1996).

The use of weights associated with individual NHANES subsamples may be inaccurate when two subsamples that do not completely overlap are used simultaneously in an analysis (NCHS 2006a). Our waist circumference analyses used only one subsample (phthalates) and are unaffected; however, HOMA analyses used both fasting and phthalate subsamples, which only overlap by 50%. Consequently, the use of phthalate weights in our HOMA analyses could affect validity of these findings. Nevertheless, use of fasting subsample weights, phthalate weights, and no weights did not substantially alter our interpretation of the HOMA results, which also demonstrated similar patterns as our waist circumference analyses. This suggests that the use of two partially overlapping subsamples did not significantly impact our findings.

## Results

Table 1 shows the median and mean phthalate levels in the U.S. population, overall and stratified by age and race/ethnicity. Exposure levels vary widely by phthalate metabolite, with MEHP having the lowest concentration among the six we analyzed, and MEP the highest. Concentrations varied somewhat by age, with greater median levels usually found in younger age groups. For all phthalate metabolites except MEHP, > 95% of subjects were at ≥ LOD; for MEHP, 80% of subjects were ≥ LOD.

Concentrations also varied by race/ethnicity. Blacks had higher levels of exposure than whites and Mexican Americans for all phthalate metabolites. Mexican Americans had somewhat higher levels than whites for MBP, MEP, and MEHP.

Table 2 shows the regression results for crude analysis (adjusting only for urinary creatinine) and fully adjusted models. In the adjusted model 1, increasing concentrations of MBzP, MEHHP, and MEOHP were statistically significantly associated with greater waist circumference, whereas concentrations of MBP, MBzP, and MEP were significantly associated with increased log HOMA. Adjusted model 2, which adjusted for renal and hepatic function, showed declines in parameter estimates, but all significant results remained so except HOMA-MBP.

Adjusted model 1 HOMA results gave similar parameter estimates whether calculated with phthalate or fasting subsample weights (< 5% difference for analyses with significant *p*-values). For further verification, an unweighted HOMA analysis was also conducted (data not shown) with somewhat larger changes in parameter estimates. However, MBP, MEP, and MBzP were still significantly associated and MEHP was again not significant. MEHHP and MEOHP gained significance in the unweighted analysis.

**Figure 1:**
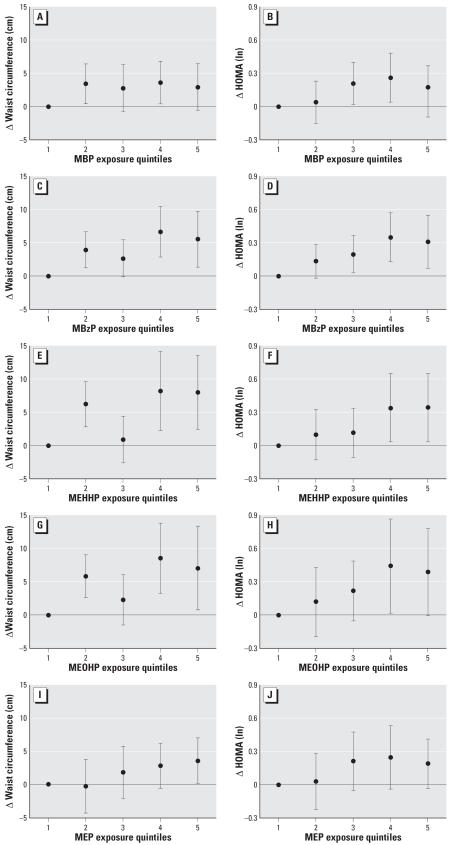
Fully-adjusted associations (adjusted model 1) between changes in outcomes and exposure quintiles (quintile 1 is reference) for metabolites with significant/near-significant (*p* ≤ 0.10) continuous regression coefficients. Error bars indicate 95% confidence intervals.

Categorical dose–response analyses demonstrated that the assumption of linearity was not strongly violated, although the curves appeared to level off, or perhaps decline, at higher metabolite concentrations (Figure 1).

The contribution of phthalate metabolites to model fit is displayed in Table 3. Adjusted *R*^2^ of the full model ranged from 15 to 20% for both outcome measures. The addition of significant phthalate metabolites explained 0.4–2.1% of outcome variability. Compared with the overall explanatory power of the full model, individual metabolites contributed between 2.5 and 10.1% of the model fit.

To convert regression coefficients to clinically interpretable measures, we computed the change in waist circumference and HOMA represented by an increase in significant phthalate metabolites from the 10th to 90th percentiles in adjusted model 1. Waist circumference increased 3.9–7.8 cm (4.0–8.0% of the 97.0-cm median) for four significant metabolites: MEP (3.9 cm), MBzP (5.8 cm), MEHHP (7.3 cm), and MEOHP (7.8 cm). HOMA (at the 2.50 median) increased 1.3–1.4 (52–57% of median) in association with three metabolites: MBP (1.3), MEP (1.3), and MBzP (1.4).

## Discussion

Obesity, insulin resistance, and diabetes have increased substantially in prevalence over the past three decades. Many plausible causes have been described, such as perinatal exposures, reduced physical activity, food marketing strategies, poor city planning, and thrifty genes (Ebbeling et al. 2002). However, if bioavailable testosterone levels in American men have declined considerably in recent years, as recently reported by Travison et al. (2007), then hypoandrogenism could be another contributor to the epidemic of obesity and related disorders. Hormonally active agents such as phthalates could be one source of this decline in testosterone level or, perhaps independently, to a decline in androgen function.

In the present study, we found that the log-transformed concentrations of several phthalate metabolites were positively and significantly correlated with abdominal obesity (MBzP, MEHHP, MEOHP, MEP) and insulin resistance (MBP, MBzP, MEP) in adult U.S. males. Categorical analysis of these metabolites by exposure quintiles yielded dose–response curves consistent with this interpretation. Although wide confidence intervals preclude strong assertions, the HOMA analyses curves suggest the inverted-U shaped, non-monotonic dose–response sometimes seen with hormonally active agents, including phthalates (Andrade et al. 2006; Lehmann et al. 2004; Takano et al. 2006; Welshons et al. 2003).

Adjustment for renal function had minimal effect. Adjustment for liver function moderated most associations but only eliminated statistical significance for HOMA-MBP. Whether this adjustment for liver function was entirely appropriate is unclear, however. Adjustment could be appropriate because obesity can affect liver function, and thus may alter phthalate metabolism. Conversely, adjusting for liver function could falsely attenuate real effects if phthalate exposure was partially responsible for elevation of liver enzymes.

Among the DEHP metabolites available, MEHP showed substantially weaker associations compared with the oxidative metabolites MEHHP and MEOHP. This was not unexpected because a recent study showed MEHHP and MEOHP more active in animal models than MEHP (Stroheker et al. 2005). Further, MEHP has a shorter serum half-life than these other DEHP metabolites, thus reducing the correlation of MEHP measurements with DEHP exposure (Koch et al. 2005).

To our knowledge, ours is the first human study to examine associations between phthalate metabolites and either abdominal obesity or insulin resistance. In one animal study, however, female rats exposed to DEHP were found to have increased serum glucose and decreased insulin, as well as thyroid and adrenocortical dysfunction (Gayathri et al. 2004). However, the decreased insulin in that study points to impaired beta cell function in the pancreas, as found in type 1 diabetes or later in the course of type 2 diabetes.

Like our study, others have found associations with outcomes that might follow from antiandrogenic effects of MBP (Duty et al. 2003; Hauser et al. 2006; Main et al. 2006; Swan et al. 2005) and MEP (Jönsson et al. 2005; Main et al. 2006; Swan et al. 2005), and found no association with MEHP (Duty et al. 2003; Hauser et al. 2006; Jönsson et al. 2005; Main et al. 2006; Swan et al. 2005). Results for MBzP, MEHHP, and MEOHP were less consistent. Assuming these associations represent true effects, differences between our results and others could be due to our larger sample sizes and increased power. Phthalates might also affect adult males differently than fetuses, or they may interact directly with energy balance or glucose metabolism pathways in addition to anti-androgenic effects.

In our study, individual phthalate exposures only explained ≤ 2.1% of the variability of our outcomes (abdominal obesity and insulin resistance). The full model, however, explained only 15–20%, despite including the well-accepted covariates of age, race/ethnicity, fat and calorie intake, activity levels, and smoking. In part, this demonstrates the difficulty of predicting the presence of this complex, multi-factorial clinical syndrome. In addition, humans are exposed to multiple phthalates simultaneously and in combination with other potential environmental toxicants that may add together to produce adverse effects. For example, PCBs, dioxins, and organochlorine pesticides have also been associated with diabetes prevalence in adult humans (Lee et al. 2006; Rylander et al. 2005). Combinations of phthalates and other potential antiandrogens have been shown in animal models to act in a dose-additive manner (Gray et al. 2006; Hotchkiss et al. 2004); in humans, Hauser et al. (2005) found greater than additive effects between MBP and PCB-153 on semen quality. Although individual phthalate exposures in humans are generally asserted to be below the no observed effect level (NOEL), combinations of man-made estrogenic chemicals, individually below NOEL concentrations, have been shown to exert considerable effect in a yeast reporter gene assay using the human estrogen receptor-α (Rajapakse et al. 2002).

Estrogenic exposures might also add to antiandrogenic exposures; some authors have found the estradiol/testosterone (total) ratio correlated more strongly with fasting glucose and insulin than testosterone (free or total) or estradiol alone (Phillips et al. 2003). Among xenoestrogens, bisphenol A (currently unmeasured in NHANES) would be important to investigate because it has recently been shown to affect insulin production in mouse pancreatic beta cells in a manner similar to that of estrogen (Alonso-Magdalena et al. 2006).

Our findings should be considered in light of several important limitations. First, as a cross-sectional study, this design cannot examine changes over time. Second, HOMA is a static measure of insulin resistance, unlike the hyperinsulinemic–euglycemic clamp, which limits its ability to detect abnormalities in insulin secretion or peripheral glucose disposal. This may have reduced our ability to observe associations. Third, NHANES 1999–2002 contains no measures of sex hormones, gonadotropins, or sex hormone-binding globulin in men, which limits our ability to examine the mechanism of action proposed in this study. Fourth, our HOMA analyses are less generalizable to the population because specially calculated sample weights for the combined use of phthalate and fasting subsamples are not available.

Fifth, our study was restricted to adult males. Children and adolescents were excluded from our study because fasting glucose and insulin were not available in NHANES 1999–2002 for subjects < 12 years of age, and because in adolescents, hormone levels vary greatly with stage of puberty. Women were excluded due to the high degree of premenopausal fluctuations in sex hormones and the change in risk profile at menopause. Also, women appear to respond differently than men to low testosterone levels; in women, low testosterone has been associated with reduced prevalence of obesity, insulin resistance, and diabetes (Ding et al. 2006; Kalish et al. 2003; Oh et al. 2002). An analysis of the adult women in this data set indeed gave dissimilar results from men (results not shown) and deserves further study.

Sixth, the one-time spot urine samples used in this study are limited measures of long-term exposure to phthalates. If phthalates are exerting antiandrogenic effects, one might expect this effect to manifest over months to years. No study has yet examined the correlation between a spot urine phthalate measurement and year-long exposure patterns. Hauser et al. (2004b), however, found that a single spot urine predicted the highest tercile of phthalate exposure over 3 months with sensitivities from 0.56 to 0.67 and specificities from 0.83 to 0.87 among the phthalates in our study. Hauser et al. also noted that urine creatinine may not be the best way to correct for variation in urine dilution, and that specific gravity (unavailable in NHANES) may be a better approach. These flaws in exposure classification also reduced our ability to observe associations.

Seventh, people with obesity or insulin resistance may be exposed to more phthalates than people who do not have these conditions. A few Food and Drug Administration–licensed medications incorporate phthalates to modify drug delivery (Schettler 2006), and this exposure can sometimes be quite large (Hauser et al. 2004a). Because people with disease are also more likely to take medications, some of the associations we have observed, particularly with MBP and MEP (metabolites of DBP and diethyl phthalate), may be falsely strengthened through reverse causality. Adjustment for this possible confounder would be difficult, however. Phthalates are considered inert ingredients, and we are not aware of a comprehensive database of inert ingredients for prescription and over-the-counter medications, herbals, and vitamins.

The present study has several important strengths, based primarily on strengths of NHANES data. Our sample was large, nationally representative, and multiethnic. Biomarkers were used for both exposures and outcomes, and a number of relevant covariates could be controlled.

Although we based our study on the premise that phthalates are acting as anti-androgens, the relationships between phthalate metabolites, abdominal obesity, and insulin resistance may be complicated by other mechanisms. For example, phthalates can act as thyroid hormone receptor antagonists (Sugiyama et al. 2005). Some phthalate metabolites are also known to interact with peroxisome proliferator–activated receptors (PPARs), which are not only important regulators of lipid and glucose homeostasis (Evans et al. 2004) but also mediate some effects of phthalates on testicular and hepatic function (Corton and Lapinskas 2005; Lapinskas et al. 2005). Activation of PPARs appear to have generally beneficial effects on lipid and glucose homeostasis, though medications that activate PPARγ improve insulin sensitivity while simultaneously increasing nonvisceral fat mass (Semple et al. 2006).

Several research paths would help determine the importance of our findings. Animal studies conducted in human exposure ranges, as well as human cross-sectional studies, could explore the capacity of combinations of hormonally active agents to create metabolic disturbances such as those we have examined here. Studies similar to ours could be extended to women, children, and adolescents, or to related conditions such as metabolic syndrome. Measurement of sex hormones, unavailable for males in NHANES 1999–2002, might suggest possible mechanisms. Examination of lipid effects could help tease out PPAR contributions. Ultimately, longitudinal studies will be required to provide more definitive answers.

In conclusion, in this large national cross-sectional sample, several phthalate metabolites showed statistically significant positive correlations with abdominal obesity and insulin resistance in adult U.S. males. If confirmed by longitudinal studies, these associations would suggest that phthalates, a widely used family of chemicals, may contribute to the prevalence of obesity, insulin resistance, and related clinical disorders. Because phthalates are rapidly metabolized, unlike PCBs and other persistent organic contaminants, such confirmation could prompt effective actions to reduce phthalate exposure in the population.

## Figures and Tables

**Table 1:** Mean median phthalate serum metabolite concentrations (μg/g creatinine): NHANES 1999–2002.^a^

	4-Year					2-Year		
		MBP	MBzP	MEHP	MEP		MEHHP	MEOHP
	No.	Mean ± SE (median)	Mean ± SE (median)	Mean ± SE (median)	Mean ± SE (median)	No.	Mean ± SE (median)	Mean ± SE (median)
All	1,443	33.8 ± 1.6 (21.2)	29.4 ± 2.7 (14.2)	11 ± 1.3 (3.8)	771 ± 66.7 (188.1)	780	65.8 ± 7.9 (19.6)	38.7 ± 4.5 (13.2)
By age (years)								
19–35	469	36.1 ± 2.9 (23.9)	35.7 ± 6.0 (17.0)	12.8 ± 2.2 (5.1)	699.2 ± 117.2 (212.4)	253	77.5 ± 18.2 (22.1)	45.8 ± 9.9 (15.6)
36–50	386	31.0 ± 2.1 (21.4)	25.0 ± 1.8 (14.9)	13.4 ± 2.7 (3.9)	672.1 ± 130.3 (173.5)	219	68.9 ± 12.7 (20.1)	39.3 ± 8.2 (13.2)
51–65	284	33.4 ± 3.2 (18.3)	25.1 ± 4.3 (11.8)	7.1 ± 1.3 (2.5)	1017.5 ± 160.8 (208.7)	160	52.8 ± 14.4 (16.3)	32.1 ± 7.7 (11.6)
66–80	231	37.9 ± 5.7 (18.4)	25.9 ± 4.3 (11.3)	5.9 ± 1.0 (2.2)	933.7 ± 182.7 (139.1)	118	42.8 ± 17.5 (11.5)	26.0 ± 8.8 (7.5)
≥ 81	73	24.2 ± 2.9 (19.0)	49.6 ± 31.9 (12.1)	3.7 ± 0.7 (2.4)	503.7 ± 180.9 (94.8)	30	20.3 ± 4.9 (13.2)	13.5 ± 3.3 (8.3)
By ethnicity								
Mexican American	335	33.1 ± 4.1 (23.2)	24.3 ± 3.3 (12.7)	8.6 ± 1.1 (4.0)	717.0 ± 82.2 (259.6)	161	34.0 ± 7.7 (15.6)	20.6 ± 4.2 (10.8)
Other Hispanic	75	39.7 ± 6.9 (24.2)	29.3 ± 4.1 (13.8)	14.9 ± 4.5 (6.9)	1347.3 ± 338.0 (292.1)	35	48.8 ± 17.3 (25.3)	30.0 ± 10.5 (15.9)
White	717	32.0 ± 1.9 (19.0)	28.5 ± 3.3 (14.0)	10.9 ± 1.8 (3.3)	703.1 ± 72.9 (158.8)	415	70.2 ± 11.8 (19.5)	41.9 ± 6.3 (13.1)
Black	271	45.6 ± 4.4 (31.7)	44.0 ± 9.4 (19.9)	13.4 ± 3.0 (5.3)	1086.5 ± 177.1 (362.6)	150	83.0 ± 24.7 (32.1)	42.5 ± 9.0 (18.7)
Other/multiethnic	45	28.7 ± 3.9 (28.1)	18.8 ± 2.8 (11.9)	5.0 ± .8 (3.8)	295.8 ± 65.6 (84.6)	19	14.7 ± 3.4 (12.3)	9.7 ± 2.1 (9.2)

a 2001–2002 only for MEHHP and MEOHP.

**Table 2:** Association between waist circumference, HOMA (ln), and selected phthalate metabolites (ln): NHANES 1999–2002.

	Crude analysis		Adjusted model 1^a^		Adjusted model 2^b^	
Outcome	β (SE)	*p*-Value	β (SE)	*p*-Value	β (SE)	*p*-Value
Waist circumference						
1999–2002 (*n* = 1,451 crude, 1,292 adjusted)						
MBP	1.39 (0.51)	0.011	0.98 (0.50)	0.059	0.79 (0.47)	0.106
MBzP	1.18 (0.47)	0.017	1.29 (0.34)	0.001	1.09 (0.36)	0.005
MEHP	0.24 (0.40)	0.550	0.62 (0.44)	0.170	0.53 (0.42)	0.217
MEP	0.95 (0.32)	0.005	0.77 (0.29)	0.013	0.66 (0.31)	0.041
2001–2002 (*n* = 781 crude, 696 adjusted)						
MEHHP	1.82 (0.58)	0.007	1.71 (0.56)	0.008	1.65 (0.50)	0.005
MEOHP	2.00 (0.63)	0.006	1.81 (0.60)	0.009	1.79 (0.55)	0.005
HOMA (ln)						
1999–2002 (*n* = 651 crude, 622 adjusted)						
MBP	0.061 (0.024)	0.016	0.064 (0.024)	0.011	0.043 (0.023)	0.081
MBzP	0.059 (0.027)	0.037	0.079 (0.023)	0.002	0.061 (0.022)	0.009
MEHP	0.035 (0.023)	0.143	0.031 (0.025)	0.225	0.016 (0.024)	0.526
MEP	0.067 (0.021)	0.004	0.056 (0.020)	0.008	0.044 (0.021)	0.045
2001–2002 (*n* = 344 crude, 327 adjusted)						
MEHHP	0.054 (0.029)	0.078	0.055 (0.028)	0.064	0.038 (0.023)	0.126
MEOHP	0.066 (0.031)	0.052	0.060 (0.032)	0.076	0.044 (0.027)	0.125

a Adjusted for age, age^2^, race/ethnicity, total fat and calorie intake, physical activity level, smoking exposure, and urine creatinine.

b Adjusted for model 1 covariates plus GFR, ALT, and GGT.

**Table 3:** Outcome variation explained by and contribution to fit of phthalate metabolites significantly (*p* ≤ 0.05) associated with one or both outcome measures (adjusted model 1).

	Adj *R*^2^ of full model with metabolite	Adj *R*^2^ of full model without metabolite	Outcome variation explained by metabolite (%)^a^	Model contribution of metabolite (%)^b^
Waist circumference				
MBzP	0.1587	0.1517	0.7	4.4
MEHHP	0.2042	0.1837	2.1	10.0
MEOHP	0.2043	0.1837	2.1	10.1
MEP	0.1556	0.1517	0.4	2.5
HOMA (ln)				
MBP	0.1650	0.1575	0.8	4.5
MBzP	0.1748	0.1575	1.7	9.9
MEP	0.1727	0.1575	1.5	8.8

Adj, adjusted.

a (*R*^2^_with metabolite_ − *R*^2^_without metabolite_) × 100%.

b [(*R*^2^_with metabolite_ − *R*^2^_without metabolite_)/*R*^2^_with metabolite_] × 100%.
