# Erratum

**Published:** 2007-09

In the article “Concentrations of Urinary Phthalate Metabolites Are Associated with Increased Waist Circumference and Insulin Resistance in Adult U.S. Males” [ Environ Health Perspect 115:876–882 (2007)] by Stahlhut et al., the title of Table 1 incorrectly referred to “serum” metabolite concentrations instead of “urinary.” The correct title of Table 1 is “Mean and median urinary phthalate metabolite concentrations (μg/g creatinine): NHANES 1999–2002.”

The authors regret the error.

